# Single‐cell multi‐omics analysis of the tumour microenvironment for colorectal cancer liver metastasis

**DOI:** 10.1002/ctm2.70626

**Published:** 2026-03-04

**Authors:** Jiao Guan, Mingwei Guo, Min Hu, Shilin Wu, Jin Qiu, Xinran Ma, Yingying Guo, Haibing Chen, Zunqiang Zhou

**Affiliations:** ^1^ Department of Surgery Shanghai Sixth People's Hospital Affiliated to Shanghai Jiao Tong University School of Medicine Shanghai China; ^2^ Shanghai Key Laboratory of Regulatory Biology, Institute of Biomedical Sciences, School of Life Sciences East China Normal University Shanghai China; ^3^ Department of Endocrinology and Metabolism, Shanghai Tenth People's Hospital School of Medicine, Tongji University Shanghai China; ^4^ Joint Center for Translational Medicine Fengxian District Central Hospital Shanghai China

1

Dear Editor:

Colorectal cancer liver metastasis (CRLM) is the primary cause of poor patient prognosis of colorectal cancer (CRC). The tumour microenvironment (TME) undergoes complex and dynamic remodelling during the metastasis. However, the cellular heterogeneity, regulatory mechanisms and crosstalks remain unclear. In this study, we integrated scRNA‐seq, scTCR/BCR‐seq and scATAC‐seq to comprehensively characterized the cellular composition, immune dynamics and epigenetic regulation within the TME from CRC to CRLM (Figure 1A).

**FIGURE 1:**
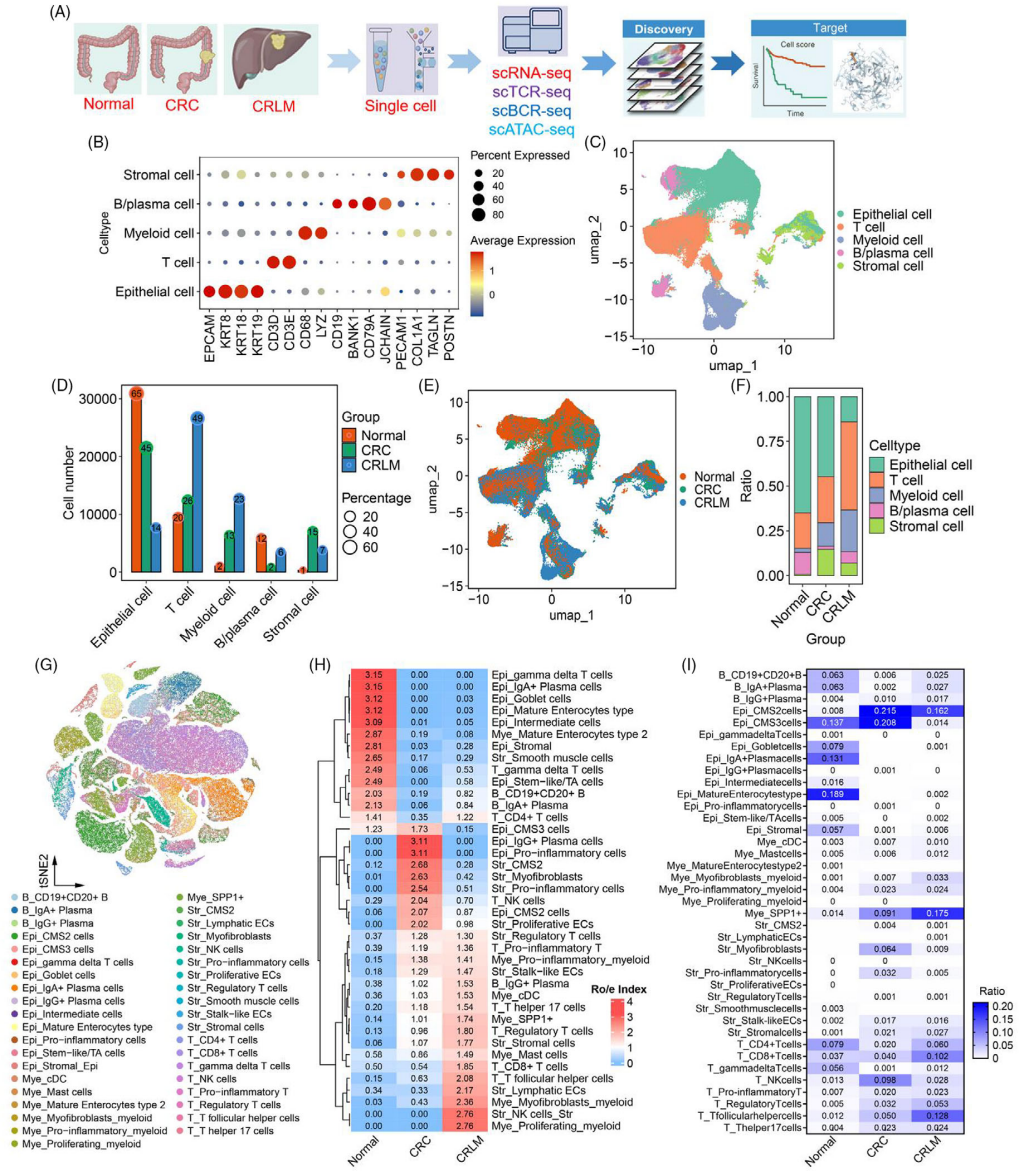
Single‐cell multi‐omics study of colorectal cancer (CRC) and liver metastasis (CRLM). (A) Flowchart illustrating the study design and workflow: Tissues from normal colorectum, primary colorectal cancer (CRC) and liver metastases (CRLM) were collected from four patients (two females and two males) for multi‐omics sequencing (scRNA‐seq, scTCR‐seq, scBCR‐seq, scATAC‐seq). Survival curve analysis was performed to evaluate the association between cell score and survival time. (B) Dot plot depicting cell type‐specific marker gene expression. In the plot, the size of each dot represents the percentage of cells expressing the gene within a specific cell type, while the colour intensity indicates the average expression level of the gene. (C) UMAP clustering plot visualizing dimensionality‐reduced single‐cell transcriptomic data, with distinct colours representing different cell types. (D) Bar graph showing the number and proportional distribution of cells across groups. The *x*‐axis represents cell types, the *y*‐axis represents cell counts and the numbers indicate the percentage of cells within each category. (E) UMAP clustering plot illustrating the cellular distribution across different disease states, with distinct colours representing different disease groups. (F) Bar chart demonstrating changes in cell type proportions across different groups. (G) UMAP plot displaying the clustering distribution of all cells, with different colours representing distinct cell types. (H) Heatmap illustrating the relative abundance changes of different cell types across normal, CRC (colorectal cancer) and CRLM (colorectal liver metastasis) groups. The Ro/e Index was used to demonstrate the enrichment level of each cell type under different conditions. (I) Heatmap showcasing the proportional differences of each cell type across three tissue states: Normal, CRC and CRLM. This provides insights into how the composition of cell types varies with disease progression from a normal state to colorectal cancer and its liver metastasis.

Cell subpopulations were annotated based on canonical marker genes reported previously.^1^ Among them, five major cell types were identified and characterized by quantitative analysis across each group tissues (Figure 1B–F, Figure S1A,S1B). Subclustering and annotation of each major cell types based on celltypist database^2^ and 40 distinct fine‐grained cell subpopulations were identified (Figure 1G, Figure S1C). Ro/e index^3^ and cell proportion analysis revealed relative subpopulation abundance across group tissues (Figure 1H,I). Of note, CD8^+^ T cells, SPP1^+^ cells, IgG^+^ Plasma cells were increased in CRC and pronounced expansion in CRLM (Figure 1H,I and Figure S1D–S1G).

Next, single cell BCR sequencing analysis revealed reduced B cell clone types and numbers, while increased clonal expansions in CRC and CRLM, indicating a significant reduction of B cell clonality in the tumour microenvironment (Figure 2A,B and Figure S2A–S2E). The somatic hypermutation (SHM) analysis, which typically reflect higher affinity BCRs,^4^ revealed that VDJ (Variable (V)‐Diversity (D)‐Joining (J)) and VJ (Variable (V)‐Joining (J)) regions in BCRs were significantly lower in CRLM compared to normal and CRC samples (Figure 2C). Besides, clonal diversity indexes including Chao1, D50, True diversity and downsampling analysis were significantly decreased in CRC and CRLM compared to normal tissues (Figure 2D, Figure S2F). BCR clonal subtypes analysis revealed reduced IGHA isotype in CRC and CRLM, while the IGHG isotype was significantly increased (Figure 2E). Further analysis of immunoglobulin isotype proportions showed an increased IGHG2 in CRC and CRLM (Figure 2F,G). These results were confirmed in an independent single‐cell cohort (Figure 2H, Figure S2G), as well as immunohistochemistry (IHC) assay on normal, CRC and CRLM samples, which showed significantly accumulated IgG^+^ plasma cells in CRC and CRLM (Figure 2I).

**FIGURE 2:**
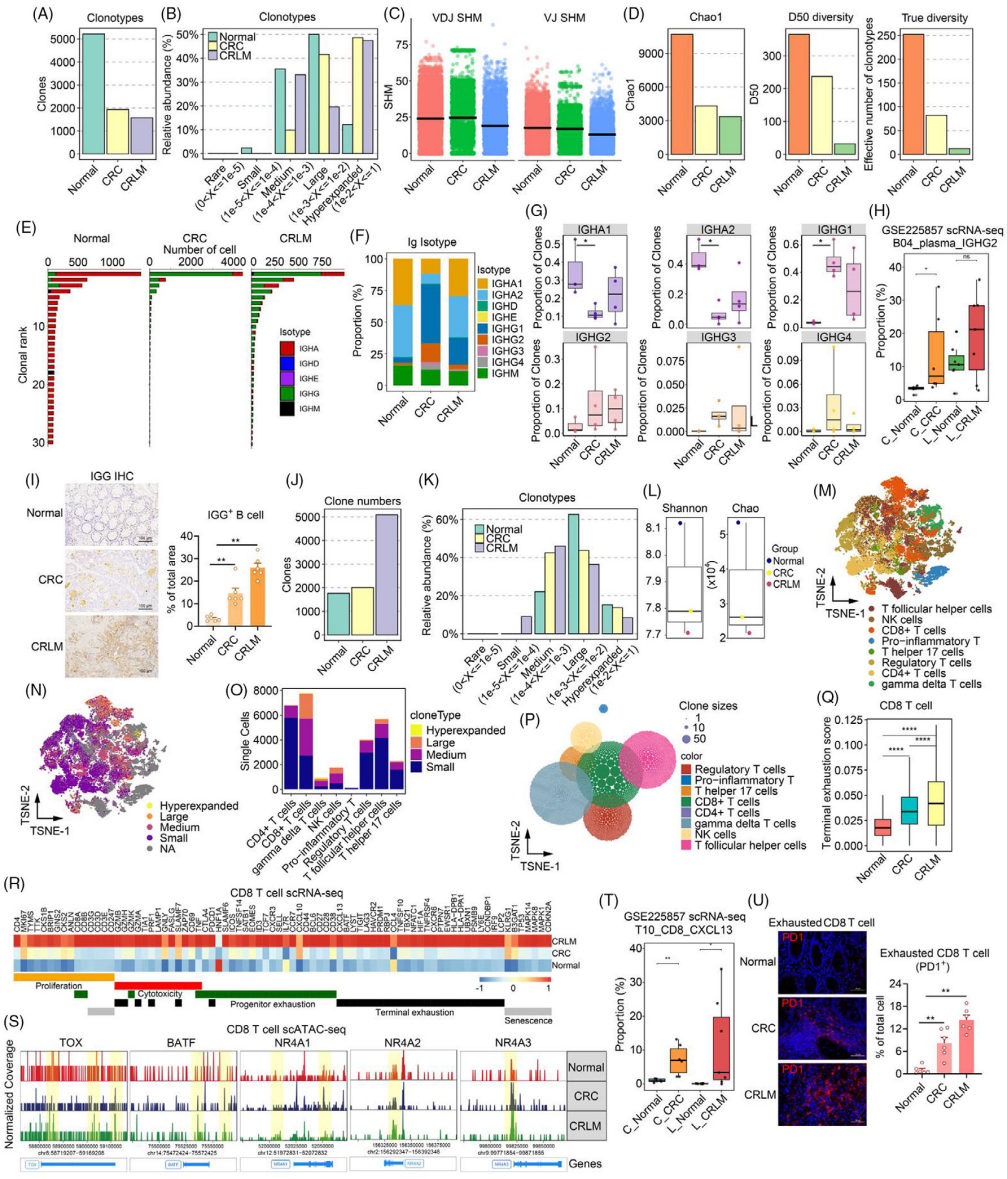
Single‐cell multi‐omics analysis of B cell receptor (BCR) and T cell receptor (TCR) clonotypes. (A) Bar chart displaying the number of B cell clones in normal, CRC (colorectal cancer) and CRLM (colorectal liver metastasis) tissues. (B) Bar charts illustrating the relative abundance distribution of different clone sizes within the immunoglobulin repertoire. (C) Scatter plot showing the levels of somatic hypermutation (SHM) in VDJ (Variable (V)‐Diversity (D)‐Joining (J)) and VJ (Variable (V)‐Joining (J)) regions across different groups. (D) Bar charts presenting diversity indices Chao1 (H), D50 (I), and true diversity (J) comparisons between different groups. (E, F) Bar charts indicating the ranking of clone numbers (E) and the distribution ratio of Ig isotypes (F) among different groups. (G) Box plot showing changes in the proportion of clones for different Ig heavy chain isotypes across all groups. (H) Box plot showing changes in the proportion of IGHG2 plasma cell (corresponding to IgG^+^ B cells) in GSE225857 scRNA‐seq across all groups. C_Nomral = Colon Normal, C_CRC = Colon CRC, L_Nomral = Liver Normal, L_CRLM = Liver CRLM. (I) Representative immunohistochemistry (IHC) picture showing the IGG^+^ B cell among different groups. *n* = 6 per group. (J) Bar chart showing the number of T cell clones in normal, CRC (colorectal cancer) and CRLM (colorectal liver metastasis) tissues. (K) Bar chart illustrating the relative abundance distribution of T cell clones across different clone sizes. (L) Box plot comparing Shannon and Chao1 diversity indices among different tissue types. (M) tSNE plots visualizing the distribution of T cell subsets, including CD8+ T cells, regulatory T cells (Tregs), follicular helper T cells (Tfh) and others. (N, O) tSNE plot showing the spatial distribution of clonal sizes across T cell populations (N), and bar chart quantifying the distribution of clonal sizes within different T cell subsets (O). (P) Bubble plot depicting the clonal size distribution across different T cell subsets, with bubble size representing clone abundance and colour indicating clonal expansion level. (Q) Box plot comparing terminal exhaustion scores among different CD8^+^ T cell subsets across normal, CRC and CRLM conditions. (R) Heatmap displaying the expression profiles of key genes associated with functional states‐including proliferation, cytotoxicity, precursor exhaustion, terminal exhaustion and senescence‐across distinct T cell subsets. (S) Normalized chromatin accessibility coverage tracks (gene body and ± 2 kb flanking regions) for marker genes TOX, BATF, NR4A1, NR4A2 and NR4A3 across different groups. Yellow shaded regions indicate the genomic locations of these genes, with higher signal peaks reflecting increased chromatin accessibility. (T) Box plot showing changes in the proportion of CD8_CXCL13 T cell (corresponding to exhausted CD8 T cells) in GSE225857 scRNA‐seq across all groups. C_Nomral = Colon Normal, C_CRC = Colon CRC, L_Nomral = Liver Normal, L_CRLM = Liver CRLM. (U) Representative immunofluorescence (IF) picture showing the exhausted CD8^+^ T cell (PD1^+^) among different groups. *n* = 6 per group.

CDR3 sequences in immunoglobulin isotypes showed that IGHG2 was significantly longer in CRC and CRLM than those in normal tissues (Figure S2H,S2I). Analysis of other types of immunoglobulin isotype showed remodelling of clonal selection and expansion, selective changes in CDR3 sequence features and amino acid physicochemical properties among different groups (Figure S2J–S2M). Subsequently, scTCR‐seq analysis found that clonal numbers in CRC and CRLM was significantly increased (Figure 2J). Analysis of clonotype relative abundance within each patient group showed that clonotypes shifted across different groups (Figure 2K, Figure S3A,S3B). Shannon, Chao diversity indexes score and downsampling analysis revealed that normal tissues had the highest diversity, following by CRC and CRLM (Figure 2L, Figure S3C). The distribution of CDR3 lengths was similar across the three tissue types, yet a slightly higher proportion of longer CDR3 lengths was observed in CRLM (Figure S3D). The constant regions, joint regions and variable regions of TRA, TRB isotype analysis showed comparable proportions among different groups (Figure S3E,S3F). Meanwhile, T cell subtype dimensionality reduction analysis demonstrated a significant increase in CD8^+^ T cells in CRC and CRLM (Figure 2M, Figure S3G). STARTRAC analysis^3^ showed that CD8^+^ T, NK cell and gamma delta T cells have higher clonal expansion, cross‐tissue migration and state transition scores (Figure S3H). Clonal type distribution among T cell subpopulations showed that hyperexpanded and large clonal types and accounted for a higher proportion in CD8^+^ T cells (Figure 2N,2O). Clonal size in T cell subpopulations revealed that CD8^+^ T cells formed the largest clones as shown in tSNE plot (Figure 2P), suggesting more pronounced clonal expansion and functional differentiation of CD8^+^ T cell in CRLM states. Analysis of TRBV and TRAV gene combinations and amino acid chemical properties indicated high special pattern in expression of genes and amino acid distributions (Figure S3I,S3J). Scoring analysis revealed higher progenitor exhaustion and terminal exhaustion scores in the CRC and CRLM groups (Figure 2Q, Figure S3K). CD8^+^ T cells exhibited heightened proliferation, cytotoxicity, exhaustion and senescence by gene expression analysis in CRC and CRLM (Figure 2R). Besides, integrated scRNA‐seq and scATAC‐seq analysis identified exhaustion‐associated TFs, including TOX, BATF, NR4A1, NR4A2, NR4A3, exhibited differential accessibility in CRC and CRLM compared with normal samples (Figure 2S), which was validated in an independent single‐cell cohort (Figure 2T, Figure S3L). In addition, we experimentally confirmed accumulated PD1^+^ exhausted CD8^+^ T cells in CRC and CRLM with immunofluorescence (IF) assay (Figure 2U).

To further explore cell‐specific epigenomic changes, we analysed single‐cell ATAC‐seq analysis among groups (Figure S4A–S4E). UMAP analysis showed significant differences in chromatin accessibility among various cell types (Figure 3A,B). The number and distribution of open chromatin peaks revealed that most accessible regions were enriched in distal regulatory regions, introns and promoter areas, whereas exons have the least representation, suggesting non‐coding regulatory elements may primarily participate in gene expression regulation (Figure 3C). The heatmap of differential chromatin accessibility peaks indicated unique chromatin accessibility patterns across cell types (Figure S4F). The cell type specific peaks enrichment analysis showed enhanced accessibility, which reaffirming the activation of cell‐specific gene regulatory networks and specific expression footprint at the single‐cell level (Figure 3D,E and Figure S4G,S4H). Integrated scATAC‐seq and scRNA‐seq analysis identified higher transcription factor openness correlates with increased gene expression (Figure 3F,G). Enrichment and key regulatory factors pseudotime analyses on open peak distributions indicated epithelial and myeloid cells were increased in CRC and CRLM, suggesting gene suppression in T cells during tumorigenesis (Figure 3H,I).

**FIGURE 3:**
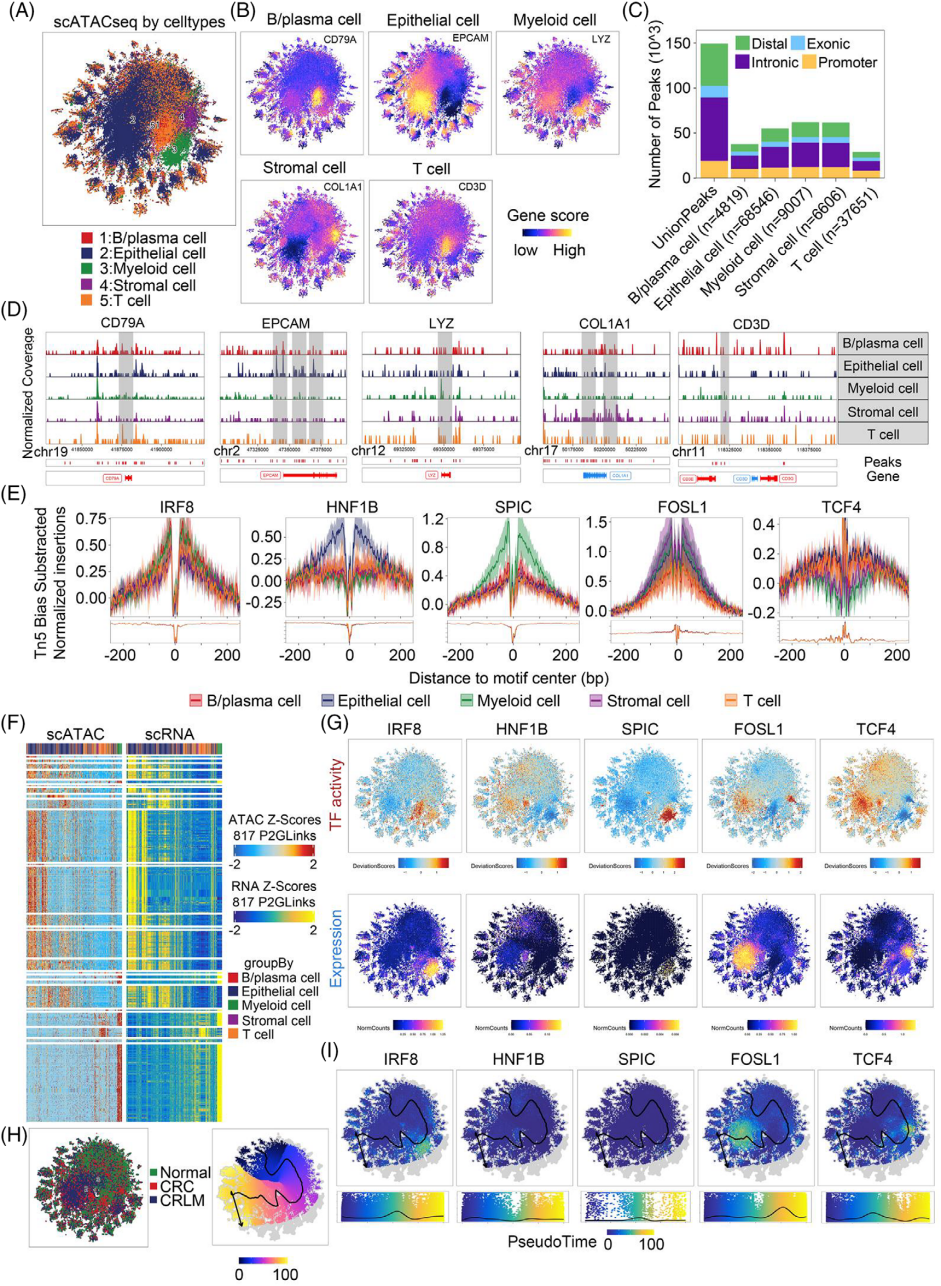
Integrated analysis of scATAC‐seq and scRNA‐seq data reveals chromatin accessibility. (A) tSNE plot displaying the clustering of all cells based on chromatin accessibility (scATAC‐seq), with different colours representing the five major cell types. (B) tSNE plots showing the gene activity scores of key marker genes‐CD79A, EPCAM, LYZ, COL1A1 and CD3D‐within each cell type. The colour intensity represents the level of chromatin openness, which was highly correlated with cellular identity. (C) Bar chart illustrating the genomic distribution of regulatory peaks across different cell types, categorized into promoter, exonic, intronic and distal intergenic (enhancer) regions. (D) Normalized chromatin accessibility coverage tracks (gene body and ± 2 kb flanking regions) for marker genes CD79A, EPCAM, LYZ, COL1A1 and CD3D in different cell types. Grey shaded regions indicate the genomic locations of these genes, with higher signal peaks reflecting increased chromatin accessibility. (E) Transcription factor footprinting analysis revealing subpopulation‐specific TFs with high regulatory activity, inferred from differential motif accessibility within accessible chromatin regions. (F) Heatmap comparing integrated scATAC‐seq (ATAC Z‐scores) and scRNA‐seq (RNA Z‐scores) data across 817 P2GLinks (peak‐to‐gene links), demonstrating coordinated regulatory relationships between chromatin accessibility and gene expression. (G) tSNE plots showing the spatial distribution of transcription factor activity (top row) and corresponding gene expression levels (bottom row) at single‐cell resolution for IRF8, HNF1B, SPIC, FOSL1 and TCF4, illustrating concordance or divergence between regulatory potential and transcript abundance. (H) Left panel: tSNE plot depicting cellular distribution from normal, CRC and CRLM samples. Right panel: pseudotime trajectory inference map, with colour gradients indicating dynamic changes along transcription factor‐driven paths, reflecting the evolutionary trends of cellular states during tumour progression. (I) tSNE plots (top) and corresponding pseudotime trajectory expression profiles (bottom) showing the dynamic expression patterns of IRF8, HNF1B, SPIC, FOSL1 and TCF4 across the inferred developmental trajectory, revealing how key transcription factors were sequentially activated or suppressed during disease progression.

Furthermore, we performed CellChat analysis to predict cell‐cell interactions across distinct cell types (Figure S5A–S5E). Notably, compared to normal tissues, both CRC and CRLM exhibited significant increases in multiple collagen family proteins, particularly with communications from stromal cells to T cells (Figure 4A, Figure S6A). Overlapping ligand‐receptor pairs in patients revealed that COL4A2‐CD44 was the top pair in CRLM than in CRC (Figure 4B). Functional enrichment analysis showed that genes up‐regulated in cancer associated fibroblasts (CAFs) were significantly enriched in the collagen chain trimerization pathway (Figure S6B), while genes down‐regulated in CAFs were significantly enriched in T cell functional pathways, including T cell receptor signalling pathway, CTLA4 signalling pathway and cytotoxic activity‐related pathways (Figure S6C–S6E), Further analysis revealed increased expression of COL4A2 in CAF and CD44 in T cells of CRC and CRLM samples (Figure 4C). Besides, SCENIC transcription factor analysis^5^, ^6^ in CAF identified a of key transcriptional regulators TFE3 (Figure 4D). RNA‐seq identified TFE3 and COL4A2 were co‐upregulated in CRLM (Figure S6F). Correlation analysis in our *in house* and additional dataset (GSE225857, GSE41568, GSE107422, GSE50760, GSE39582) showed positive correlation between *TFE3* and *COL4A2* expression levels (Figure 4E, Figure S6G,S6H). Indeed, we confirmed that TFE3 overexpression in CAFs increased COL4A2 mRNA and protein levels, while TFE3 knockdown in CAFs decreased COL4A2 mRNA and protein levels (Figure 4F,G). Besides, scATAC‐seq and TFE3 Chip‐seq data (GSE246789) revealed significantly increased chromatin accessibility at promoter regions of *COL4A2* (Figure 4H,I). Furthermore, in silico analysis revealed a TFE3 binding site ‐CACGCG‐ at positions ‐1072/‐1066 upon *COL4A2* promoter (Figure 4I). Indeed, luciferase reporter assay showed that TFE3 overexpression dose‐dependently increased *COL4A2* promoter activity, while these effects were blunted with TFE3 binding site mutation (Figure 4J,K). Finally, TCGA data analysis demonstrated that CRC patients with high expression of both TFE3 and COL4A2 showed worse prognosis for OS (overall survival) and RFS (recurrence‐free survival) outcomes in TCGA CRC cohorts as showed in Kaplan‐Meier curves (Figure 4L, Figure S6I).

**FIGURE 4:**
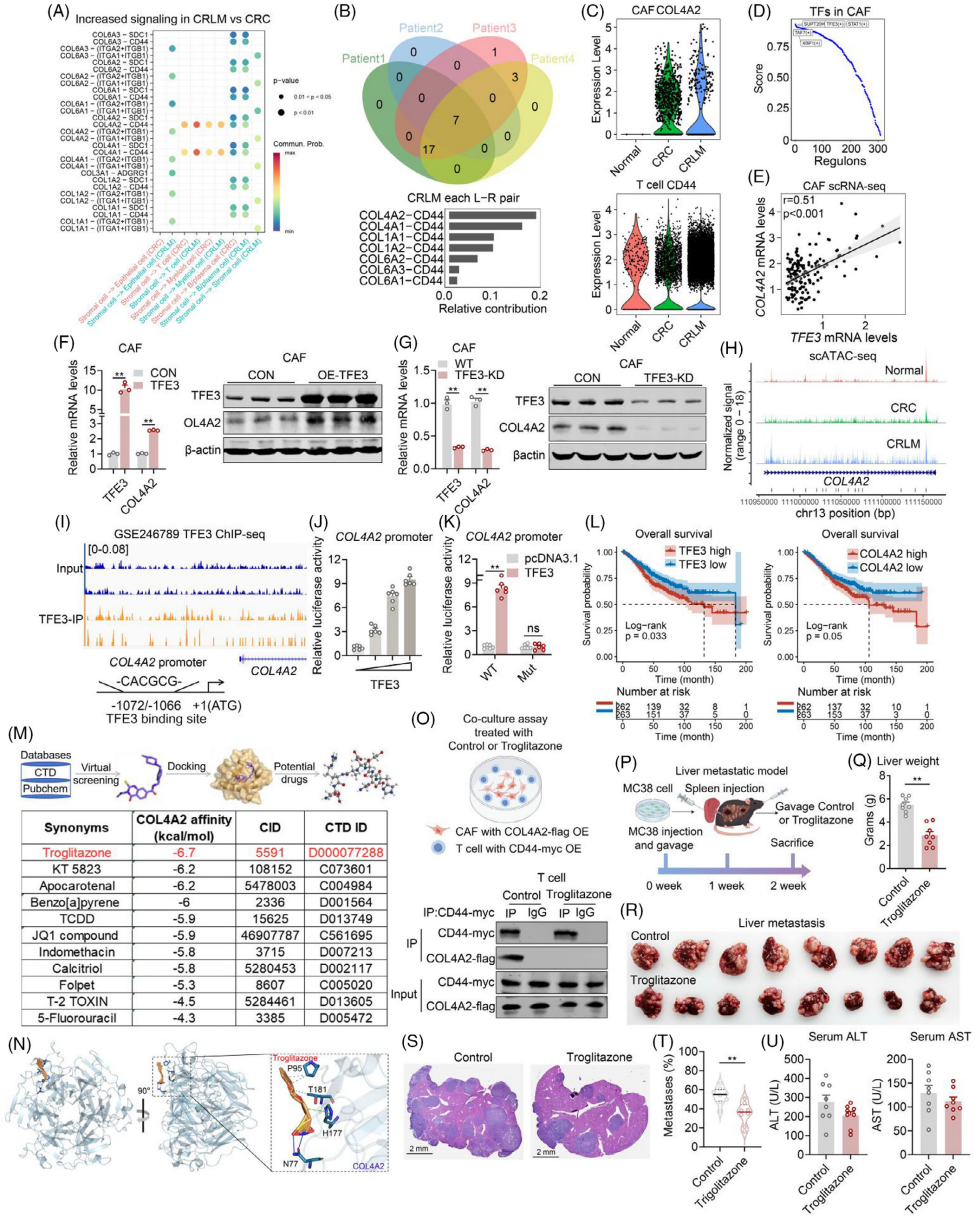
TFE3 activates COL4A2 expression in cancer‐associated fibroblasts (CAFs) mediates communications with T Cells. (A) Bubble charts illustrating intercellular signalling communications between distinct cell types. The pathways more active in CRLM compared to CRC. Each bubble represents a specific ligand receptor pair, with size and colour intensity reflecting the magnitude of interaction. (B) Venn diagram showing the overlap of ligand–receptor pairs in intercellular communication networks among four individual patients. (C) Violin plots displaying the expression levels of COL4A2 in CAFs across Normal, CRC, and CRLM tissues (left), and CD44 expression levels in T cells across the same conditions (right). (D) Line plot showing the ranked enrichment scores of key transcription factors (TFs) in cancer‐associated fibroblasts (CAFs), with TFE3 exhibiting one of the highest regulatory potential scores. (E) Correlation scatter plot demonstrating a significant positive correlation between TFE3 and COL4A2 expression levels in CAFs. qPCR and Western blot analyses showing TFE3 and COL4A2 expression in CAFs with TFE3 overexpression. (G) qPCR and Western blot analyses showing TFE3 and COL4A2 expression in CAFs with TFE3 knockdown. (H) Genomic signal track displaying chromatin accessibility changes at the COL4A2 locus across normal, CRC and CRLM tissues. Increased accessibility in CRC and particularly in CRLM implies enhanced regulatory activity at this locus during tumour progression. (I) Peak plot showing the TFE3 binding site in TFE3 ChIP‐seq data of GSE246789 and potential binding sites CACGCG (‒1072/‒1066) in COL4A2 promoter as shown in schematic diagram. (J) Luciferase assay assessing the dose dependent regulation TFE3 on COL4A2 transcription. *n *= 6 biological replicates. (K) Luciferase assays depicted the relative luciferase activity of specific TFE3 binding site deletion on COL4A2 promoters under the regulation of TFE3. WT and Mut CACGCG (‒1072/‒1066) of WT. *n* = 6 biological replicates. (L) Kaplan‐Meier survival curves showing that patients with high expression of TFE3 and COL4A2 have significantly lower overall survival compared to those with low expression. (M and N) Molecular docking and structural analysis reveal potential small‐molecule regulatory targets of COL4A2. (M) Table listing small‐molecule compounds with high binding affinity (measured in kcal/mol) to COL4A2. Among these, troglitazone exhibits the highest binding affinity at ‐6.7 kcal/mol. (N) Molecular docking model depicting the binding mode of troglitazone to COL4A2 analysed by PLIP. Blue solid line indicates hydrogen bonding interactions with O‐H bond and key residues involved in the binding include Asn77(N77). Green dashed lines represent π‐π and key residues involved in the binding include His177(H177). Grey dashed lines represent hydrophobic effect and key residues involved in the binding include Thr181(T181), and Pro95(P95). (O) Schematic diagram showing coculture assay between CAF with COL4A2‐flag OE and T cell with CD44‐Myc OE under Control or Troglitazone treatment and Co‐IP analysis in T cell. (P‐U) Analysis of phenotypes of CRLM animal model treated with 0.5% carboxymethylcellulose sodium (CMC‐Na) solution or Troglitazone (10 mg/kg/d) five times a week for 2 weeks gavage administration. (P) Schematic diagram showing liver metastatic model; (Q) Liver weight; (R) Liver metastasis; (S) H&E staining; (T) Tumour numbers; (U) Serum ALT and AST tests in CRLM animal models treated with 0.5% CMC‐Na or Troglitazone (10 mg/kg/d) five times a week for2 weeks gavage administration. *n* = 8 per group.

For further therapeutic purposes, we employed a virtual screening strategy to identify small‐molecule compounds via CTD database that potentially interacted with COL4A2 (Figure 4M). Interestingly, after ranking the compounds based on their binding affinity scores to COL4A2, the top candidate was Troglitazone, one of the well‐known anti‐diabetic and anti‐cancer drugs. Molecular docking analysis showed that troglitazone exhibited the strongest predicted binding affinity (–6.7 kcal/mol) (Figure 4M), Specifically, troglitazone formed a hydrogen bond with Asn77(A) and engaged in hydrophobic/π‐stacking interactions with Tyr185(A), His177(A), Arg179(A), Thr181(A) and Pro95(A) of COL4A2, stabilizing its position in the binding pocket (Figure 4N, Figure S6J).

We have previously predicted that CAFs may secret COL4A2, which interacted with receptor CD44 in CD8^+^ T cell for exhaustion. Thus, we performed co‐culture analysis between CAFs overexpressed with COL4A2 and CD8^+^ T cells overexpressed with CD44. Then, Co‐IP analysis was performed in CD8^+^ T cells treated with or without Troglitazone. Of note, we found that COL4A2 interacted with CD44, while the binding was blocked under troglitazone treatment (Figure 4O). In addition, we established an in vivo colorectal liver metastatic model treated with or without troglitazone. The results showed that troglitazone significantly blocked colorectal liver metastasis, suggesting that troglitazone may be effective in the inhibition of CRLM (Figure 4P–T), without possible hepatotoxicity, as shown by serum ALT and AST levels (Figure 4U). These data supported its potential to inhibit COL4A2 function and influence extracellular matrix integrity in tumour progression.

In summary, by integrating single‐cell multi‐omics technologies, we present the comprehensive atlas of TME evolution during CRC progression from primary to metastatic sites and systematically delineate the multi‐layered remodelling of cellular composition, immune clonality, epigenetic regulation and intercellular communication, which reveal that TFE3‐COL4A2 axis in CAFs communicated with CD44 in T cells for exhaustion in CRLM, which might be blocked by troglitazone (Figure S6K).

## AUTHOR CONTRIBUTIONS

Zunqiang Zhou, Haibing Chen and Yingying Guo conceived and designed the experiments. Jiao Guan collected human samples and processed patients’ clinical information. Jiao Guan, Mingwei Guo and Xinran Ma performed bioinformatic analysis. Mingwei Guo, Min Hu, Shilin Wu, Jin Qiu and Yingying Guo performed the molecular and animal experiments and analysed the data. Xinran Ma and Mingwei Guo drafted the manuscript. Zunqiang Zhou, Haibing Chen and Yingying Guo supervised the work and revised the manuscript. All authors read and approved the manuscript.

## CONFLICT OF INTEREST STATEMENT

The authors declare no conflicts of interest.

## FUNDING INFORMATION

This work was supported by funds from National Key Research and Development Program of China (2024YFA0917700), National Natural Science Foundation of China (32325024, 82301777), The Postdoctoral Fellowship Program of CPSF (GZB20230219), China Postdoctoral Science Foundation (2023M741183). The ECNU Public Platform for Innovation (011) and the Instruments Sharing Platform of School of Life Science.

## ETHICS STATEMENT

This study was approved by the Institutional Ethics Committee of Shanghai Sixth People's Hospital, Shanghai Jiao Tong University (Approval No: 2024‐YS‐317). All procedures performed in tasks involving human participants were under the ethical standards of the institutional research committee. All participants have provided written informed consent prior to sample collection.

## Supporting information



Supporting Information

Supporting Information

Supporting Information

Supporting Information

## Data Availability

The data that support the findings of this study are available contact the corresponding author.

## References

[1] Liu X , Wang X , Yang Q , et al. Th17 cells secrete TWEAK to trigger epithelial–mesenchymal transition and promote colorectal cancer liver metastasis. Can Res. 2024;84:1352‐1371. doi:10.1158/0008‐5472.CAN‐23‐2123 10.1158/0008-5472.CAN-23-2123 38335276

[2] Domínguez Conde C , Xu C , Jarvis LB , et al. Cross‐tissue immune cell analysis reveals tissue‐specific features in humans. Science. 2022;376:eabl5197. doi: 10.1126/science.abl5197 35549406 10.1126/science.abl5197 PMC7612735

[3] Zhang L , Yu X , Zheng L , et al. Lineage tracking reveals dynamic relationships of T cells in colorectal cancer. Nature. 2018;564:268‐272. doi:10.1038/s41586‐018‐0694‐x 30479382 10.1038/s41586-018-0694-x

[4] Mesin L , Ersching J , Victora Gabriel D . Germinal center B cell dynamics. Immunity. 2016; 45: 471‐482. doi:10.1016/j.immuni.2016.09.001 27653600 10.1016/j.immuni.2016.09.001 PMC5123673

[5] Aibar S , González‐Blas CB , Moerman T , et al. SCENIC: single‐cell regulatory network inference and clustering. Nat Method. 2017;14:1083‐1086. doi:10.1038/nmeth.4463 10.1038/nmeth.4463 PMC5937676 28991892

[6] Van de Sande B , Flerin C , Davie K , et al. A scalable SCENIC workflow for single‐cell gene regulatory network analysis. Nat Protocol. 2020;15:2247‐2276. doi:10.1038/s41596‐020‐0336‐2 10.1038/s41596-020-0336-2 32561888

