# Supplementary material for: Single‐cell multi‐omics analysis of the tumour microenvironment for colorectal cancer liver metastasis
Source: Clin Transl Med. 2026 Mar 4;16(3):e70626. doi: 10.1002/ctm2.70626 (PMC12960060; doi:10.1002/ctm2.70626)
Supplement: Supplementary file 1 — Supporting Information [file CTM2-16-e70626-s001.docx]

**Supplementary Table S1. The comprehensive patient tumor profiles**

| Primary Tumor Site | Primary Tumor Size (cm^3^) | Histological Type | MLH1 | MSH2 | MSH6 | PMS2 | Ki67 |
| --- | --- | --- | --- | --- | --- | --- | --- |
| Sigmoid Colon | 4.5*3.5*1.5 | Tubular Adenocarcinoma Grade II-III | + | + | + | + | 80%+ |
| Rectum | 5.5*5.0*4.5 | Tubular Adenocarcinoma Grade II-III | + | + | + | + | 80%+ |
| Sigmoid Colon | 5.0*4.0*2.8 | Tubular Adenocarcinoma Grade II-III | + | + | + | + | 80%+ |
| Colon | 4.0*3.5*1.0 | Tubular Adenocarcinoma Grade II-III | + | + | + | + | 80%+ |
