# Supplementary material for: Single‐cell multi‐omics analysis of the tumour microenvironment for colorectal cancer liver metastasis
Source: Clin Transl Med. 2026 Mar 4;16(3):e70626. doi: 10.1002/ctm2.70626 (PMC12960060; doi:10.1002/ctm2.70626)
Supplement: Supplementary file 2 — Supporting Information [file CTM2-16-e70626-s004.docx]

**Supplementary Table S2. The primer sequences used in this research.**

| Gene clone primers list | |
| --- | --- |
| Primer | Sequence (5'-3') |
| h*COL4A2*-WT-F | CTGGCCTAACTGGCCGGTACCAGCTGCCTCCAGGGAGATAA |
| h*COL4A2*-WT-R | AGTACCGGATTGCCAAGCTTTTACACCGAAGGGTCCATGC |
| h*COL4A2*-Mut-F | GCCCAGAGAATGCACCTGGCCGTGCCGACCCCCGAGGGGCAGGCGGACGGG |
| h*COL4A2*-Mut-R | CCCGTCCGCCTGCCCCTCGGGGGTCGGCACGGCCAGGTGCATTCTCTGGGC |
|  |  |
| qPCR primers list. | |
| Primer | Sequence (5'-3') |
| h*GAPDH*-F | AAGACGGGCGGAGAGAAACC |
| h*GAPDH*-R | CGTTGACTCCGACCTTCACC |
| h*TFE3*-F | GATCATCAGCCTGGAGTCCAGT |
| h*TFE3*-R | AGCAGATTCCCTGACACAGGCA |
| h*COL4A2*-F | GGATAACAGGCGTGACTGGAGT |
| h*COL4A2*-R | CTTTGCCACCAGGCAGTCCAAT |
