# Supplementary material for: Single‐cell multi‐omics analysis of the tumour microenvironment for colorectal cancer liver metastasis
Source: Clin Transl Med. 2026 Mar 4;16(3):e70626. doi: 10.1002/ctm2.70626 (PMC12960060; doi:10.1002/ctm2.70626)
Supplement: Supplementary file 3 — Supporting Information [file CTM2-16-e70626-s002.docx]

**Supplementary Methods**

**scRNAseq analysis**

Fresh tissue was digested with collagenase/trypsin at 4 °C for 30 minutes, filtered through a 70 μm cell strainer, and resuspended as a single-cell suspension with >85% viability, as determined by trypan blue staining and cell counting. Cells were first subjected to flow cytometry (FACS) to remove doublets and dead cells, then diluted to a concentration of 700–1200 cells/μL. Single-cell libraries were generated using the 10x Genomics Chromium platform with GEM (Gel Bead-in-Emulsion) microfluidics. Within each GEM, cell lysis was performed using 0.5% Triton X-100, and oligo-dT primers containing Unique Molecular Identifiers (UMIs) and cell barcodes were used to capture polyadenylated mRNA and synthesize cDNA. The cDNA was amplified, fragmented, end-repaired, adenylated, and ligated with sequencing adapters prior to sequencing on the Illumina NovaSeq 6000 platform. Raw sequencing data in FASTQ format were aligned to the human reference genome (GRCh38), and the 10x Genomics Cell Ranger pipeline was used to generate the raw feature-barcode matrix. Quality control was performed by filtering cells based on the following criteria: median number of UMIs per cell>500, mitochondrial read percentage < 10%, ribosomal read percentage < 5%, and number of detected genes > 200. Seurat [3] was used for subsequent scRNAseq analysis. A Seurat object (Seurat v5.3 in R) was created, and initial filtering was applied using FilterCells (min.features = 200, max.mito.percent = 10). Normalization was performed using SCTransform, followed by selection of 3,000 highly variable genes via SelectIntegrationFeatures. Data were scaled using ScaleData, principal component analysis (PCA) was conducted to estimate significant principal components, and dimensionality reduction was performed using UMAP and t-SNE. For clustering and annotation, cell neighborhoods were identified using FindNeighbors, and clusters were detected with FindClusters at a resolution range of 0.6. Cell type annotation was initially performed using Celltypist [4], followed by manual validation against canonical marker genes from the CellMarker database. Batch effects across multiple samples were corrected using Harmony. Differential gene expression analysis for each cluster was performed using FindMarkers with the MAST test (logFC.threshold = 0.25, FDR < 0.05). Functional enrichment analysis was conducted using clusterProfiler [5] for GO and KEGG pathways. Module scores for functional characterization of cell subpopulations were calculated using AddModuleScore. Gene Set Enrichment Analysis (GSEA) was performed using the MSigDB Hallmark gene sets [6, 7] to assess biological functions such as T cell cytotoxicity and extracellular matrix (ECM) organization.

**CellChat data analysis**

CellChat analysis begins with a normalized gene expression matrix and cell annotations [8]. The CellChat object was constructed using createCellChat and populated with a species-specific signaling database (CellChatDB.human). Significantly overexpressed ligand-receptor (LR) pairs were identified using identifyOverExpressedGenes and identifyOverExpressedInteractions. The communication probabilities between cell groups were then inferred based on a mass-action model via computeCommunProb, with low-confidence interactions filtered out by setting a minimum cell threshold (min.cells=10). The interaction networks were aggregated into "number" and "strength" matrices using aggregateNet. Network visualization was performed using functions such as netVisual_circle, netVisual_chord, and netVisual_heatmap to generate circular plots, heatmaps, and chord diagrams, illustrating global or pathway-specific signaling flows. To analyze signaling at the pathway level, multiple LR pairs were integrated into pathway-level communications using computeCommunProbPathway. Network centrality measures were computed via netAnalysis_computeCentrality to identify dominant or influential cell populations within the intercellular communication network. In multi-condition studies, the above pipeline was independently applied to each dataset, followed by merging the individual CellChat objects using mergeCellChat. Inter-group differences in cellular communication were then assessed using compareInteractions and rankPathways, enabling comparative analysis of interaction patterns and pathway activities. The results were visualized as bubble plots, differential chord diagrams, and gene expression violin plots, facilitating comprehensive visualization and biological interpretation of intercellular communication networks derived from single-cell transcriptomic data.

**scATAC-seq data analysis**

scATAC-seq data analysis were begin with fragment files and single-cell peak count matrices from 10x Genomics using ArchR [9]. The createArrowFiles function was used to generate Arrow files, during which low-quality cells with a transcription start site (TSS) enrichment score below 5% were automatically filtered out. Subsequently, the reference genome was loaded via addArchRGenome, and addGroupCoverages computes pseudoreplicated coverage tracks based on user-defined cell group annotations. High-confidence peak sets were generated using addReproduciblePeakSet, which performs peak calling with MACS2 independently within each cell group and then takes the intersection of resulting peaks. A peak-by-cell count matrix was constructed using addPeakMatrix, while addGeneScoreMatrix projects chromatin accessibility signals onto gene bodies to generate a gene activity matrix. For dimensionality reduction and initial clustering, addIterativeLSI (Latent Semantic Indexing) was applied, followed by addUMAP for nonlinear embedding and addHarmony to correct batch effects and define cell clusters. Cell type annotation was performed using addAnnotations, leveraging label transfer from a reference single-cell RNA-seq dataset (e.g., Seurat-based annotation) via integrative mapping. Transcription factor (TF) footprinting analysis was conducted with addDeviationsMatrix to compute motif deviation scores as implemented in chromVAR, reflecting TF regulatory activity. Peak-to-gene annotations were assigned using peakAnnotation, linking peaks to their nearest genes and enabling downstream functional enrichment analysis. Putative cis-regulatory interactions were inferred via addCoAccessibility and addPeak2GeneLinks, which identify co-accessible peak-gene pairs and potential regulatory linkages. When integrating with matched scRNA-seq data, addIntegratedScores combines gene activity scores with corresponding transcriptomic expression profiles for multimodal analysis. Pseudotime trajectories were inferred using addTrajectory to model dynamic changes in chromatin accessibility across cellular states. Finally, plotPDF was employed to batch-generate publication-quality visualizations, including peak heatmaps, footprinting plots, UMAP embeddings, and regulatory network diagrams, enabling comprehensive interpretation of epigenomic landscapes and gene regulatory programs at single-cell resolution.

**scTCR and scBCR Data Analysis**

The scRepertoire package was used for the analysis of single-cell T cell receptor (scTCR) and B cell receptor (scBCR) sequencing data [10, 11]. Input data were generated from 10x Genomics cellranger vdj pipelines and imported into scRepertoire for downstream processing. The functions combineTCR() or combineBCR() were applied to pair and concatenate α/β chains (for TCR) or heavy/light chains (for BCR) by barcode, while filtering out low-confidence (non-productive, productive = FALSE) and multi-chain (>2 productive chains) cells. Following object creation, clonotype abundance was quantified using quantContig(), which computes clonal metrics such as Chao1 and Shannon diversity indices. Clonal expansion and diversity were visualized at both sample and cell cluster levels using clonalQuant() (for cumulative clonotype frequency curves) and clonalDiversity() (for bar plots of diversity measures). Inter-sample clonal overlap was assessed with clonalOverlap(), which calculates similarity using Jaccard or Morisita indices. To integrate with Seurat objects, combineExpression() merges clonotype information into the Seurat object’s meta.data, enabling downstream visualization. This allows clonotypes of interest to be highlighted on feature plots using highlightClonotypes(), facilitating the correlation of clonal identity with gene expression or cell phenotype.

Alternatively, the immunarch package can be used to process 10x Genomics cellranger vdj output [12]. The repLoad() function automatically parses paired α/β or heavy/light chain sequences and removes non-functional (non-productive) or ambiguous rearrangements. repClonality function from the immunarch package, with the .clone.types parameter defining clonal size categories based on their frequency as a proportion of total reads: Rare = 1e-05 (0.001%), Small = 1e-04 (0.01%), Medium = 0.001 (0.1%), Large = 0.01 (1%), and Hyperexpanded = 1 (100%). Clonal exploration was performed via repExplore(), which calculates key repertoire metrics per sample, including the number of unique clonotypes, D50 (clonotype diversity index), Shannon entropy, and clonality curves. Downsampling analyses to compare the estimated clonal diversity of T and B cells across sample groups as a function of sequencing depth. When integrating with a Seurat single-cell object, immunarch appends the clonotype table to the meta.data slot, enabling joint analysis of clonotypic and transcriptomic data. Finally, the vis() family of functions enables one-step generation of publication-ready visualizations, including boxplots and violin plots of repertoire diversity across conditions, supporting comprehensive immune repertoire profiling at single-cell resolution.

The Platypus software package was utilized for the analysis of single-cell T-cell receptor (scTCR) and B-cell receptor (scBCR) data [13]. Somatic hypermutation (SHM) analysis was performed using VDJ_plot_SHM(). The function VDJ_clonal_donut() was employed for the visualization of clonal distribution. VDJ_Vgene_usage calculates and visualizes the usage frequency of V genes. VDJ_logoplot_vector() was used to generate a sequence logo for clonotype CDR3 sequences, showcasing the conservation and diversity of amino acids at each position.

The STARTRAC package was used to analyze clonotype-based dynamics of T cells across different tissues, including clonal expansion, distribution, migration index, and Ro/e ratio [14].

**TCGA Data Analysis**

Transcriptomic data for primary colorectal cancer (CRC) samples were retrieved using the TCGAbiolinks R package with the command GDCquery(project= "TCGA-COAD", data.category="Transcriptome Profiling", workflow.type = "HTSeq -FPKM-UQ"). Similarly, metastatic samples from both TCGA-COAD and TCGA-READ projects were queried using identical parameters to identify cases of colorectal liver metastasis (CRLM). Clinical data were downloaded via GDCquery_clinic, harmonized by standardizing field names, and filtered to exclude patients with missing follow-up times or survival durations ≤ 0 days. Gene expression levels were dichotomized into high and low expression groups based on either the median value or an optimal cut-off determined by survival analysis. Survival objects were constructed using the Surv(time, status) function from the survival package. Kaplan-Meier survival curves were fitted using survfit, and differences between groups were assessed by the log-rank test implemented in survdiff. Variables showing significant univariate associations were further evaluated in multivariate Cox proportional hazards models using coxph(), with adjustment for age, sex, tumor stage, and microsatellite instability (MSI) status. Final survival plots were generated using survminer::ggsurvplot(), producing Kaplan-Meier curves with 95% confidence bands, at-risk tables, and annotated p-values for clear and comprehensive visualization of survival differences.

**SCENIC Transcription Factor Analysis**

SCENIC analysis [15, 16] was performed using the normalized gene expression matrix from Seurat as input. First, pySCENIC's grnboost2 algorithm was applied to infer co-expression modules between each transcription factor (TF) and its potential target genes, generating putative regulatory networks (regulons) based on a gradient-boosting random forest approach. Subsequently, the cisTarget database (hg38) was used to perform motif enrichment and ranking analysis for the genes within each module. Only regulons with a Normalized Enrichment Score (NES) > 3, false discovery rate (FDR) < 0.05, and at least 20 target genes were retained as significant, thereby defining TFs and their directly regulated target genes as functionally active regulons. The motif conservation of top-ranking regulons was further validated using RcisTarget to ensure evolutionary conservation of predicted cis-regulatory elements.

**Molecular Docking**

Protein structural information for COL4A2 and CD44 was retrieved from UniProt (https://www.uniprot.org/) and the Protein Data Bank (PDB, https://www.rcsb.org/). To identify small-molecule compounds capable of modulating the expression of COL4A2 and CD44, we utilized the Comparative Toxicogenomics Database (CTD, https://ctdbase.org/), a publicly available resource. The 3D structural data of selected small molecules were downloaded from PubChem and converted from SDF to mol2 format using Open Babel. The receptor proteins were preprocessed in PyMOL by removing water molecules and co-crystallized ligands, followed by hydrogen addition and charge assignment using AutoDock Tools. Both ligands and receptors were then converted into PDBQT format for docking calculations performed with AutoDock Vina [17]. The resulting docking poses were analyzed for potential interaction profiles using the online tools PLIP (Protein-Ligand Interaction Profiler) [18] and LigPlot+ [19]. Ligand-receptor binding conformations and interaction sites were visualized in PyMOL to assess key molecular interactions, including hydrogen bonds, hydrophobic contacts, and π-stacking. This integrative approach enabled the identification and characterization of candidate compounds with potential regulatory effects on COL4A2 and CD44, supporting their evaluation as therapeutic targets.

**Human tissue samples**

Human tissue specimens, including normal colon mucosa, primary colorectal cancer (CRC), and matched colorectal liver metastases (CRLM), were prospectively collected from patients undergoing surgical resection at Shanghai Sixth People’s Hospital. Normal colon tissues were obtained from macroscopically and histologically confirmed non-neoplastic regions at least 10 cm away from the primary tumor margin. Fresh tissue samples were immediately processed after surgical excision: one portion was fixed in 10% neutral buffered formalin for 48 hours and subsequently embedded in paraffin for histopathological validation; another portion was snap-frozen in liquid nitrogen within 15 minutes of resection and stored at −80°C for molecular analyses. All procedures were performed in strict accordance with institutional protocols to minimize ischemic time (<30 minutes). The study was approved by the Institutional Ethics Committee of Shanghai Sixth People's Hospital, Shanghai Jiao Tong University (Approval No.: 2024-YS-317). All participants have provided written informed consent prior to sample collection. Written informed consent was obtained from all participants prior to sample collection. Patient clinical data were recorded comprehensive patient tumor profiles, including gender, age, primary tumor site, histological type, molecular characteristics including MLH1, MSH2, MSH6, PMS2, Ki67 as shown in Supplementary Table S1. These patients were all free from gene mutation of KRAS: Q61L(182A>T), Q61R(182A>G), Q61H(183A>C), Q61H(183A>T), K117N(351A>C), K117N(351A>T), A146T(436G>A), A146V(437C>T), A146P(436G>C); NRAS: G12D(35G>A), G12S(34G>A), G13R(37G>C), G12C(34G>T), G12V(35G>T), G12A(35G>C), G13V(38G>T), Q61R(182A>G), Q61K(181C>A), Q61L(182A>T), Q61H(183A>C), A146T(436G>A); PIK3CA: H1047R(3140A>G), H1047L(3140A>T) and BRAF: V600E(1799T>A).

**Quantitative Real-time polymerase chain reaction (qPCR)**

Total RNA was extracted from cultured cells using RNAiso Plus (9108; Takara, Kusatsu, Shiga, Japan). RNA concentration and purity were assessed using a NanoDrop (Thermo Fisher Scientific, Waltham, MA, USA), with acceptable samples showing an A260/A280 ratio of approximately 1.8-2.0. 500 ng RNA was reverse-transcribed into first-strand cDNA in a 20 μL reaction mixture using PrimeScript^TM^RT Master Mix (RR036A; Takara) according to the manufacturer’s protocol. The reaction was incubated at 37°C for 15 minutes, followed by enzyme inactivation at 85°C for 5 seconds. The resulting cDNA was diluted 10-fold and used as template for subsequent qPCR. The qPCR reactions were performed in a total volume of 10 μL, containing SYBR Green Master Mix (4309155; Thermo Fisher Scientific), gene-specific forward and reverse primers, diluted cDNA template, and nuclease-free water. Amplification was carried out on a real-time PCR system (LightCycler 480; Roche, Basel, Switzerland) with the following cycling conditions: initial denaturation at 95°C for 30 seconds, followed by 40 cycles of 95°C for 5 seconds and 60°C for 30 seconds. After amplification, relative gene expression levels were analyzed using the 2^−ΔΔCt^ method, with GAPDH used as the housekeeping gene for normalization. Primer sequences were shown in supplementary table S2.

**Coimmunoprecipitation assay (Co-IP) and Western Blot**

Cell samples were lysed on ice for 30 minutes in RIPA lysis buffer (P0013D; Beyotime Biotechnology, Shanghai, China) supplemented with protease and phosphatase inhibitors. The lysates were then centrifuged at 12,000 g for 15 minutes at 4°C, and the supernatants were collected. An appropriate volume of SDS loading buffer containing DTT was added to each sample, followed by vortexing and denaturation at 100°C for 10 minutes. Samples were immediately placed on ice after heating. Proteins were separated by SDS-PAGE and then transferred onto a nitrocellulose (NC) membrane using wet transfer at 100 V for 90 minutes. After transfer, the membrane was blocked with 5% non-fat milk in TBST (Tris-buffered saline with 0.1% Tween-20) on a rocking platform at room temperature for 1 hour to block non-specific binding sites. The primary antibody was diluted according to the manufacturer’s instructions in blocking buffer or TBST, and the membrane was incubated in this solution overnight at 4°C. Following incubation, the membrane was washed three times with TBST (10 minutes per wash) to remove unbound primary antibody. A fluorescently labeled secondary antibody was then applied, and the membrane was incubated at room temperature for 2 hour in the dark. After three additional TBST washes (10 minutes each), the membrane was imaged using a fluorescence-based imaging system. The antibodies used were as follows: Anti-Flag-Tag (F1804; Sigma-Aldrich), Anti-Myc-Tag (19C2, Abmart), Anti-COL4A2 (AF6534, Beyotime), Anti-TFE3 (14480-1-AP, Proteintech), Anti-β-Actin (sc-47778, Santa Cruz Biotechnology, Inc.).

**Cell culture**

Cells were cultured in a humidified incubator maintained at 37°C with 5% CO₂ and >95% relative humidity. 293T, CCD-18Co and MC38 cell lines were grown in high-glucose Dulbecco’s Modified Eagle Medium (DMEM, Gibco, 11995065) supplemented with 10% fetal bovine serum (FBS, Gibco, 10270) and 1% penicillin–streptomycin (100 U/mL penicillin and 100 μg/mL streptomycin, Gibco, 15070063). The suspension cell line Jurkat was routinely maintained in RPMI-1640 (Gibco, 8123414) medium, also supplemented with 10% FBS and 1% penicillin–streptomycin. Cells were passaged every 2-3 days. For adherent cells, when confluence reached 70–90%, they were detached using 0.25% trypsin-EDTA (Gibco, 25200-056), and the digestion was neutralized by adding complete growth medium containing serum. The cell suspension was then centrifuged at 300 g for 5 minutes, resuspended in fresh medium, and seeded into new culture dishes at an appropriate split ratio. Jurkat cells were maintained by direct dilution into fresh medium to a target density of 0.6 × 10⁶ cells/mL. All procedures were performed in a biosafety cabinet under strict aseptic conditions.

**Transfections and luciferase assays**

HEK293T cells (CRL-3216; ATCC) were seeded in 24-well plates at a density of 5 × 10⁴ cells per well and cultured overnight to reach 70–80% confluency. Cells were co-transfected with the indicated plasmids using the EZ-trans transfection reagent (C4058L1090; Life ilab Biotechnology, Shanghai, China), following the manufacturer’s protocol. The transfection mixture typically included: the COL4A2 promoter-driven firefly luciferase reporter construct (wild-type or mutant variants cloned into pGL4.17 vector); expression plasmids for transcription factors such as OE-TFE3, sh-TFE3, COL4A2, CD44 were purchased from a biotech company, pRL-SV40 vector expressing Renilla luciferase as an internal control for normalization. Luciferase activity was measured 24 hours post-transfection using the Dual-Luciferase® Reporter Assay System (TM040; Promega, Madison, WI, USA). Briefly, cells were lysed with Passive Lysis Buffer, and both firefly and Renilla luciferase activities were quantified sequentially on a luminometer. Firefly luciferase signals were normalized to Renilla luciferase readings to account for variations in transfection efficiency and cell viability. All experiments were performed in triplicate and repeated independently at least three times. The full-length COL4A2 promoter reporter was generated by cloning a ~2.5 kb genomic fragment upstream of the transcription start site into pGL4.17. Tthe mutated reporters were generated by deleting specific regulatory elements (CACGCG, −1072/−1066) -using overlap extension PCR (SOE-PCR). All constructs were verified by Sanger sequencing. Primer sequences are provided in Supplementary Table 2.

**Cell co-culture assay**

Intestinal myofibroblast CCD-18Co cells were used to represent cancer-associated fibroblasts (CAFs). A stable COL4A2-overexpressing cell line was generated by lentiviral transduction using a pCDH vector encoding COL4A2 with a C-terminal FLAG tag, followed by puromycin selection. For T cells, Jurkat cells were transduced with a lentivirus carrying CD44 fused to a Myc tag in a pCDH backbone and subjected to puromycin selection to establish a stable CD44-Myc–overexpressing Jurkat T cell cell line. For co-culture experiments, the two cell lines were seeded into Transwell plates (0.4 μm pore size) at a 1:1 ratio to allow paracrine signaling and membrane contact while preventing direct cell mixing. For cell treatment, control group treated with an equal volume of DMSO vehicle, and Troglitazone treated group treated with Troglitazone at a final concentration of 10 μM , with continuous exposure for 24 hours. After 48 hours of co-culture, Jurkat T cells were harvested for downstream analysis. For co-immunoprecipitation (Co-IP), total protein was extracted from the harvested CD8⁺ T-like Jurkat cells using RIPA lysis buffer supplemented with protease inhibitors. An aliquot of the lysate was reserved as an Input control and analyzed by Western blot to confirm the expression levels of CD44-Myc and COL4A2-FLAG. The remaining lysate was subjected to immunoprecipitation using an anti-Myc antibody (19C2, Abmart), with normal mouse IgG (sc-2025, SANTA CRUZ BIOTECHNOLOGY, INC) serving as a negative control. The resulting immunoprecipitates were separated by SDS-PAGE and analyzed by Western blot.

**Establishment of CRLM model and Troglitazone Treatment**

A mouse model of colorectal cancer liver metastasis was established by intrasplenic injection of MC38 cells, a method that effectively recapitulates the natural dissemination of tumor cells via the portal venous system to the liver. Briefly, 8-week-old wild-type C57BL/6 mice were used for all experiments. Under sterile conditions, mice were anesthetized using inhaled isoflurane, followed by shaving and disinfection of the left upper abdominal area. A 1 cm incision was made below the left costal margin to carefully expose the spleen. A suspension of MC38 colorectal carcinoma cells (1×10⁶ cells per mouse in 50 μL of PBS) was slowly injected into the splenic parenchyma using a 27–30 G needle, taking care to avoid deep puncture that could cause bleeding or cell leakage. After injection, gentle pressure was applied to the puncture site to achieve hemostasis, the spleen was returned to the abdominal cavity, and the peritoneum and skin were sutured layer by layer. Postoperatively, mice received analgesia and anti-inflammatory treatment and were closely monitored for recovery. For therapeutic intervention, mice were randomly assigned to two groups starting one day after surgery: Control group were administered 0.5% carboxymethylcellulose sodium (CMC-Na) solution; Treatment group were administered Troglitazone at 10 mg/kg/day, five times per week for two weeks. Troglitazone was delivered by oral gavage as a suspension freshly prepared by homogenizing or sonication in 0.5% CMC-Na solution. The control group received an equal volume of vehicle (0.5% CMC-Na) by gavage at the same time each day under identical handling conditions. At the end of the 2-week treatment period, mice were euthanized. Livers were completely excised, weighed, and visually examined. Liver tissues were fixed, paraffin-embedded, and sectioned for hematoxylin and eosin (H&E) staining. Whole-slide scanning was performed, and the number and size of metastatic lesions in liver sections were quantified histologically.

**Histomorphological Analysis**

For Immunohistochemistry (IHC) analysis, tissues were harvested immediately after surgery or euthanasia and fixed in 10% neutral buffered formalin for 48 hours. Following fixation, tissues were dehydrated through a graded ethanol series, cleared in xylene, infiltrated with paraffin, and embedded in paraffin blocks. Serial sections of 5 μm thickness were cut using a microtome and mounted onto adhesive-coated slides (e.g., poly-L-lysine or silane-treated). Slides were baked at 60°C for 2 hours to enhance tissue adhesion. Deparaffinization was performed by immersing slides in xylene (2×10 min), followed by rehydration through a descending ethanol series (100%→95%→70%→distilled water). the slides were immersed in either pH 6.0 sodium citrate buffer or pH 9.0 EDTA/Tris-EDTA buffer and heated for 10 minutes using a microwave, pressure cooker, or steamer. Slides were then allowed to cool to room temperature and washed three times (5 min each) with PBS or TBS. Endogenous peroxidase activity was blocked by incubating slides in 3% H₂O₂ at room temperature for 15 minutes. After PBS washing, sections were blocked with 5% normal goat serum at room temperature for 30 minutes to reduce non-specific binding. Primary antibody Rabbit Anti-Human IgG Polyclonal Antibody (10284-1-AP, Proteintec) was applied and incubated overnight at 4°C. Slides were washed three times (5 min each) with PBS. A horseradish peroxidase (HRP)-conjugated secondary antibody goat anti-rabbit IgG-HRP was applied and incubated at room temperature for 60 minutes. Following three additional PBS washes (5 min each), immunoreactivity was visualized using 3,3'-diaminobenzidine (DAB, ZLI-9018, ZSGB-BIO) chromogen. Nuclei were counterstained with hematoxylin, followed by dehydration through an ascending ethanol series, clearing in xylene, and mounting with neutral balsam. Stained sections were examined under a brightfield microscope.

For Immunofluorescence (IF) analysis, sample preparation up to antigen retrieval and blocking was identical to the IHC protocol described above. After blocking, primary antibody anti-PD-1 (GB113744, Servicebio) was applied and incubated overnight at 4°C. Slides were washed three times (5 min each) with PBS, with all subsequent steps performed in the dark to prevent fluorophore quenching. A fluorophore-conjugated secondary antibody Alexa Fluor® 488 goat anti-rabbit IgG (1:500) was applied and incubated at room temperature for 60 minutes in the dark. Nuclei were counterstained with DAPI (C1006, Beyotime Biotechnology) for 5 minutes at room temperature, protected from light. Slides were gently rinsed twice with PBS and mounted using an anti-fade mounting medium. Mounted slides were cured overnight in the dark. Fluorescent signals were visualized using a fluorescence or confocal microscope, with images acquired separately for each fluorescence channel. Quantitative analysis, including co-localization, fluorescence intensity measurement, and cell counting, was performed using ImageJ software.

For Hematoxylin and Eosin (H&E, C0105S, Beyotime Biotechnology, Shanghai, China) staining analysis, paraffin sections (5 mm) were cut from liver. Tissue sections were photographed using a Nikon camera (ECLIPSE Ci-Lplus, Nikon).

**Serum ALT and AST level determination**

Serum samples were analysed using commercially available enzyme-based assay kits (ALT, Sigma-Aldrich, MAK052, AST, Sigma-Aldrich, MAK055) to determine alanine aminotransferase (ALT) and aspartate aminotransferase (AST) levels, with procedures adhering to the manufacturer's instructions.

**Statistical analysis**

The data analysis was performed with R package (Version 3.63; CRAN) and GraphPad Prism10 (GraphPad Software, San Diego, CA, USA). Shapiro-Wilk normality tests were performed for all datasets to ensure that all data matched normal distribution before statistical analysis. Statistical comparisons between 2 groups were made by 2-tailed unpaired student t tests for unpaired data or by paired t tests for paired data. Correlation analysis was calculated using the Spearman’s method. Data were shown as mean±SEM or median and interquartile range. Statistical significance was set at p < 0.05.
