# Supplementary material for: Single‐cell multi‐omics analysis of the tumour microenvironment for colorectal cancer liver metastasis
Source: Clin Transl Med. 2026 Mar 4;16(3):e70626. doi: 10.1002/ctm2.70626 (PMC12960060; doi:10.1002/ctm2.70626)

Supplementary Figure S1

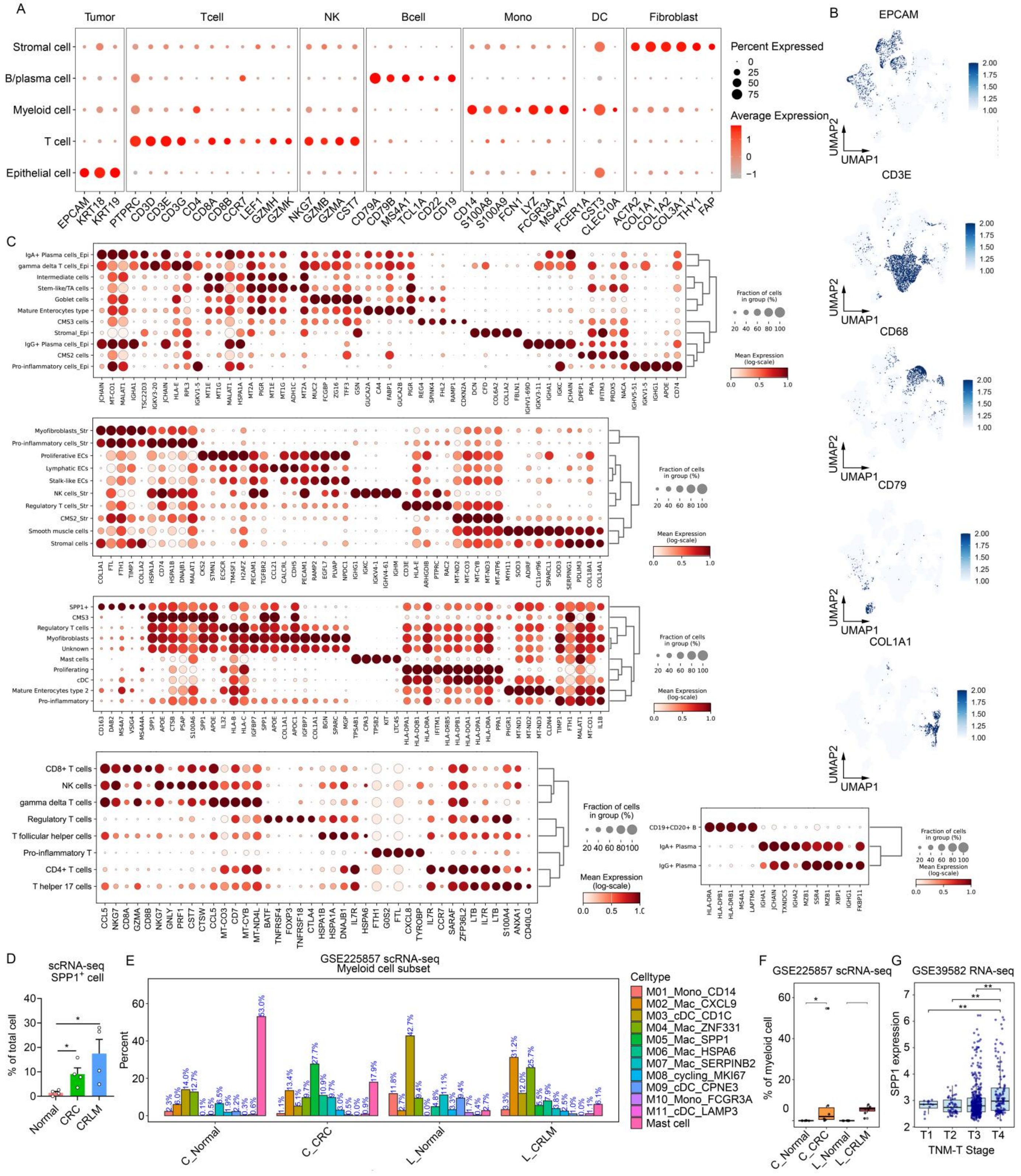

Supplementary Figure S2

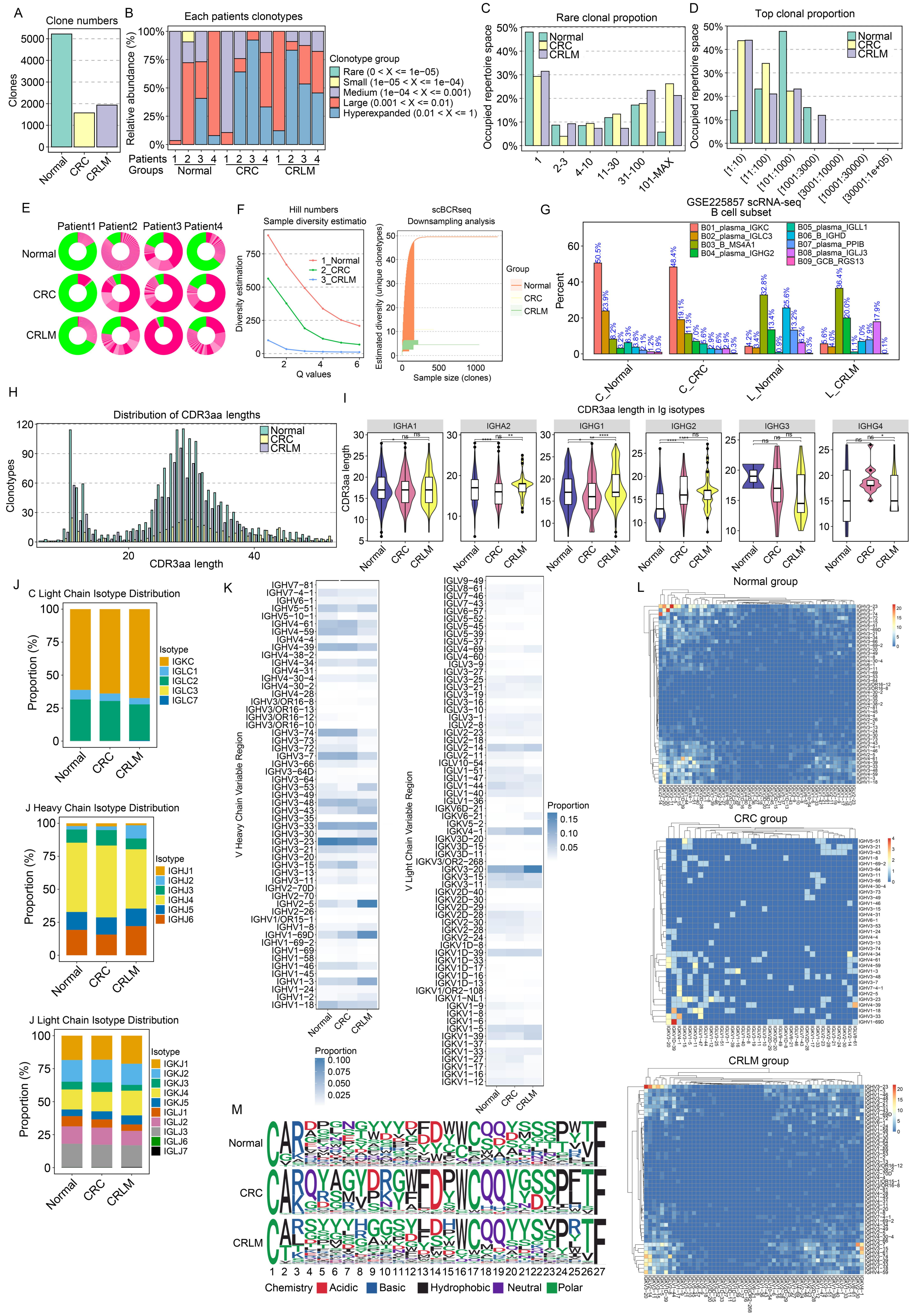

Supplementary Figure S3

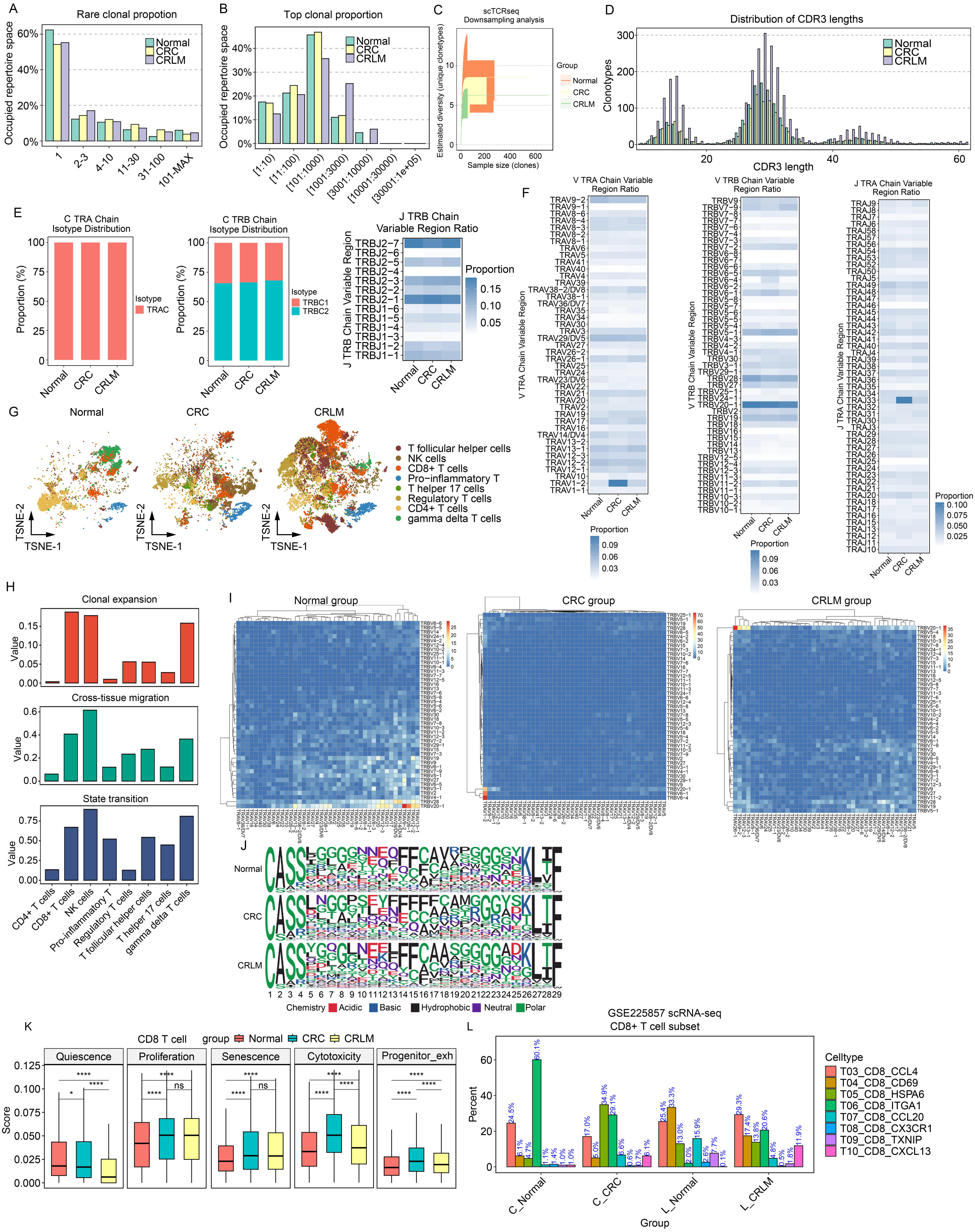

Supplementary Figure S4

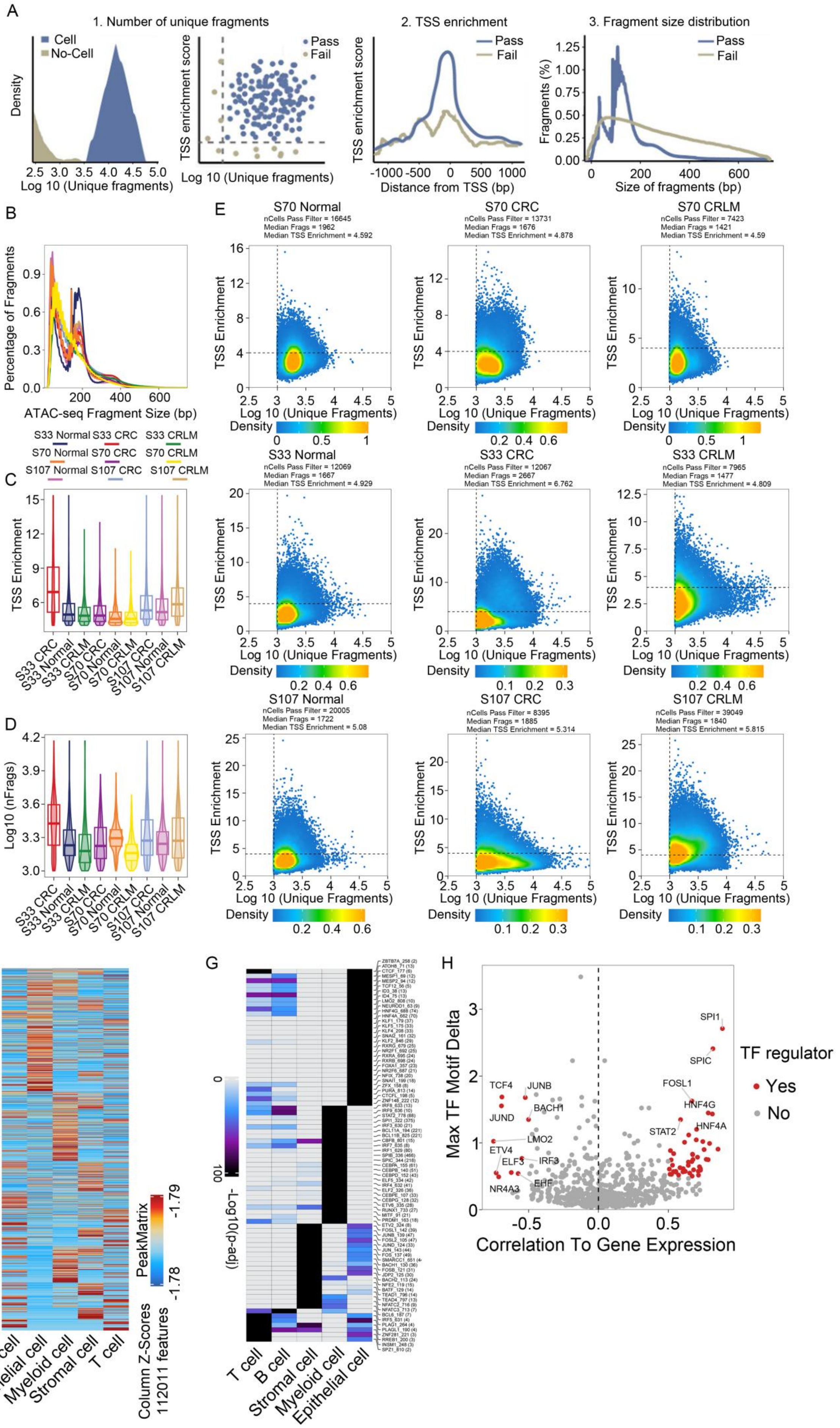

Supplementary Figure S5

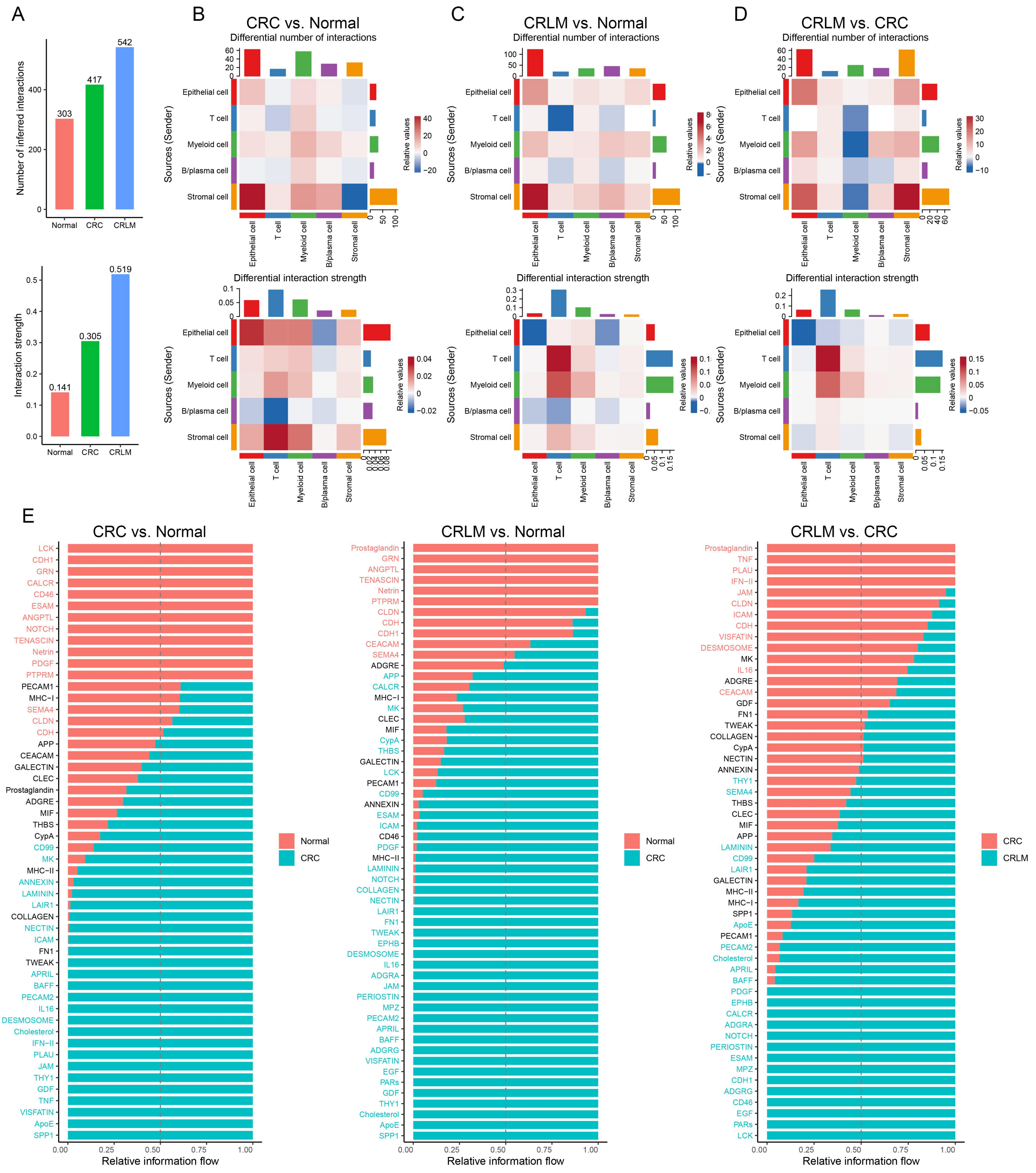

Supplementary Figure S6

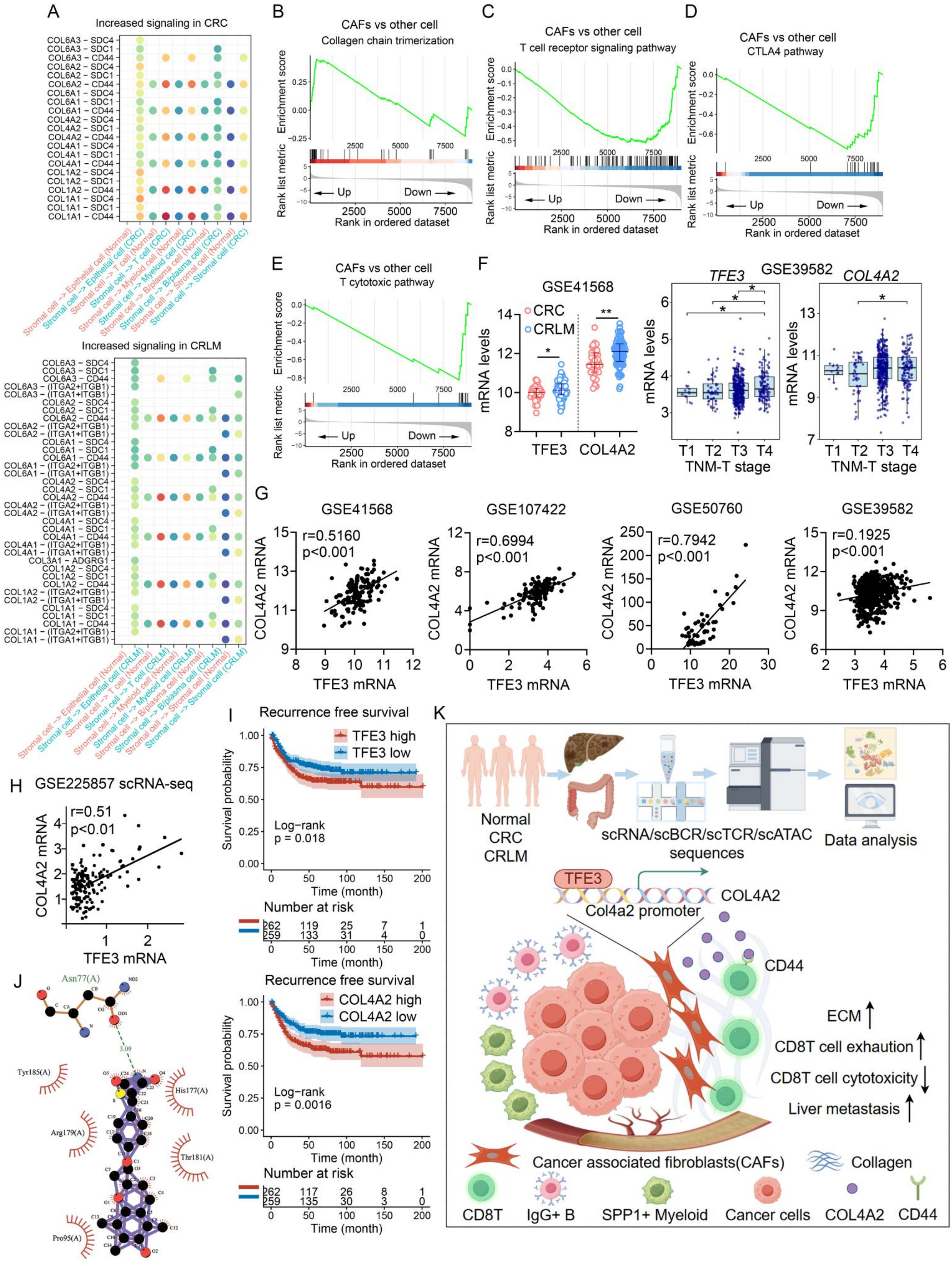

Supplement: Supplementary file 4 — Supporting Information [file CTM2-16-e70626-s003.pdf]
